# Parameter determination and transformation for the focusing of dielectric microspheres illuminated by optical needle

**DOI:** 10.1038/s41598-017-06146-7

**Published:** 2017-07-18

**Authors:** Tongnan Xia, Hanming Guo, Jinbing Hu, Songlin Zhuang

**Affiliations:** Engineering Research Center of Optical Instrument and System, Ministry of Education; Shanghai Key Lab of Modern Optical System, College of Optical-Electrical Information and Computer Engineering, University of Shanghai for Science and Technology, Shanghai, 200093 China

## Abstract

By eliminating the spherical aberrations of microsphere we derived a simple but useful formula on the focusing of dielectric microsphere. On basis of this formula, not only can researchers determine the parameters of an optical microsphere system with super-resolution, but they can also perform parameter transformation. In order to facilitate the application, the principle of parameter transformation was summarized into three kinds of case listed in Table 1, which were all demonstrated numerically with concrete examples by finite-difference time-domain method. This formula will be conducive to the development of applications based on microsphere, such as photonic nano-jet lithography, microsphere nano-scope.

## Introduction

Microspheres have recently attracted numerous attentions in the field of super-resolution due to its sub-diffraction focusing size and magnification of objects ahead of it^1–7^, and have been used in nano-scale imaging^8, 9^, nano-scale lithography^10, 11^ etc. The physical mechanism of sub-diffraction focusing size of microsphere is that microspheres have naturally negligible spherical aberrations (SAs) and high numerical aperture (NA) when the refractive index ratio (RIR) between the microsphere and its surrounding medium and the radius of microsphere are designed properly^12^. Before wide-ranging applications in practice, however, two significant issues still should be resolved:

(i) How to determine the set of parameter of an optical microsphere system to focus the incident beam at the shadow side of microsphere;
(ii) How to obtain the same resolution (normalized to the wavelength in surrounding medium) as before if one or more parameters are altered, i.e., performing parameter transformation without changing the resolution of microsphere system.

As for these issues above, few literatures concern about them. The super-resolutions by means of microspheres reported were mostly obtained with either fixed wavelength (e.g., the resolution of λ/17 in ref. 9 with the illumination wavelength of 408 nm) or specific surrounding medium (e.g., the resolution of λ/7 in the ref. 13 with the microsphere immersed in isopropyl alcohol). Authors did not show whether the same resolution could be achieved if other light source was used or the microsphere was immersed in other liquid, and how to make it.

Here, the present article aims to resolve the above issues. According to the imaging theory, low SAs and high numerical aperture (NA) are two preconditions of high resolution of microsphere^14, 15^. In our model, the focusing property of microsphere with radius in the range from *n*_1_ * λ_0_ to 1.4 * *n*_1_ * λ_0_ (*n*_1_ and λ_0_ are the refractive index of surrounding medium and illumination wavelength in free space, respectively) mainly depend on SAs; NA only makes a small effect to the focusing property of microsphere, because NA is very small (<0.1*n*_1_). Thus, the only approach obtaining high resolution in our model is eliminating SAs of microsphere. As reported in ref. 12, microspheres possess positive and negative SAs; the positive SAs is inversely proportional to RIR, while the negative SAs is closely related to the wavelength-scale dimension of microsphere; hence, we offset SAs by adjusting RIR and the radius of microsphere. Once SAs are well eliminated the incident beam will be focused at the shadow side of microsphere, and the highest resolution is achieved for the microsphere system. More importantly, a simple but useful formula among the refractive index of surrounding medium, the illumination wavelength, the radius and refractive index of microsphere was derived. On the basis of this formula, not only can researchers design an optical system of microsphere with high resolution, but they can also perform parameter transformation to the existing microsphere system, which will be conducive to applications based on microspheres, such as microsphere nano-scope^8^, photonic nano-jet lithography^11^.

## Results

In this paper, ray tracing method and finite-difference time-domain (FDTD) method are used. First, it is necessary to indicate that the validity of ray tracing method in dealing with the imaging of small-Fresnel-number system with radius λ < r < 10λ has been proved theoretically^16, 17^ and experimentally^18^. As reported in ref. 16 that for nonconventional system, defined as with characteristic dimension λ < r < 10λ, the results obtained by ray tracing approach are in excellent agreement with electromagnetic theory (see Table 1 in ref. 16), and the same thesis was obtained by ref. 18 in the experimental investigation of imaging of circular aperture with wavelength-scale radius.

**Table 1:** Principles of parameter transformation by eq. (4).

Notes	Laser wavelength	Surrounding index	Microsphere index	Microsphere radius
Original parameters	λ_0_	*n*_1_	*n*_2_	*r*
I	ηλ_0_^a^	*n*_1_	*n*_2_	η*r*
II	λ_0_	η*n*_1_^a^	η*n*_2_	η*r*
III	σλ_0_^a^	η*n*_1_^a^	η*n*_2_	ση*r*

^a^Denotes active parameter, others are passive parameters.

**Figure 1:**
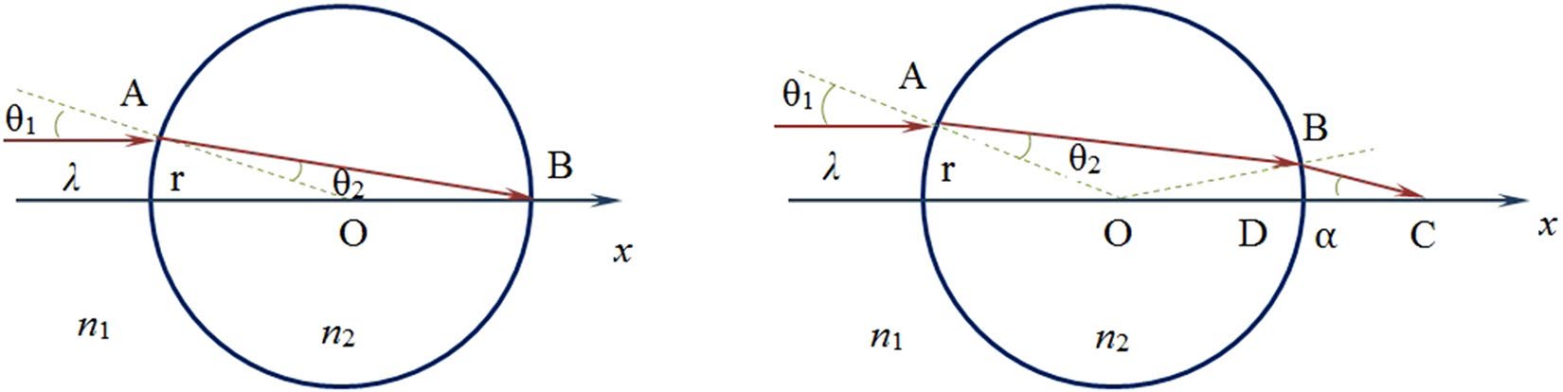
Schematic of a microsphere illuminated by optical needle with width of 0.43λ_0_.

In our model, the optical needle with width of 0.43λ_0_, which is generated by tightly focusing of a radially polarized Bessel-Gaussian (BG) beam with a combination of a binary-phase element and a high-numerical-aperture lens^19–21^, was used as illumination source. As shown in left panel of Fig. 1, the optical needle is incident on microsphere from left to right, i.e., along *x* axis. The radius and refractive index of microsphere are *r* and *n*_2_, respectively. *θ*_1_, *θ*_2_ are incident angle and refraction angle, respectively, satisfying Snell’s law: *n*_1_∙sin*θ*_1_ = *n*_2_∙sin*θ*_2_. The surrounding medium has refractive index of *n*_1_. Obeying the ray tracing process, to focus the incident ray at the shadow-side point B, the following relationship can be extracted:

1 $${n}_{2}=2{n}_{1}\,\cos (0.215{\lambda }_{0}/2r)$$

where the value in bracket is in radian unit. Equation (1) reveals that, to focus the incident optical needle at the shadow-side point B, the refractive index ratio between the microsphere and its surrounding medium should not be larger than 2^22^.

**Figure 2:**
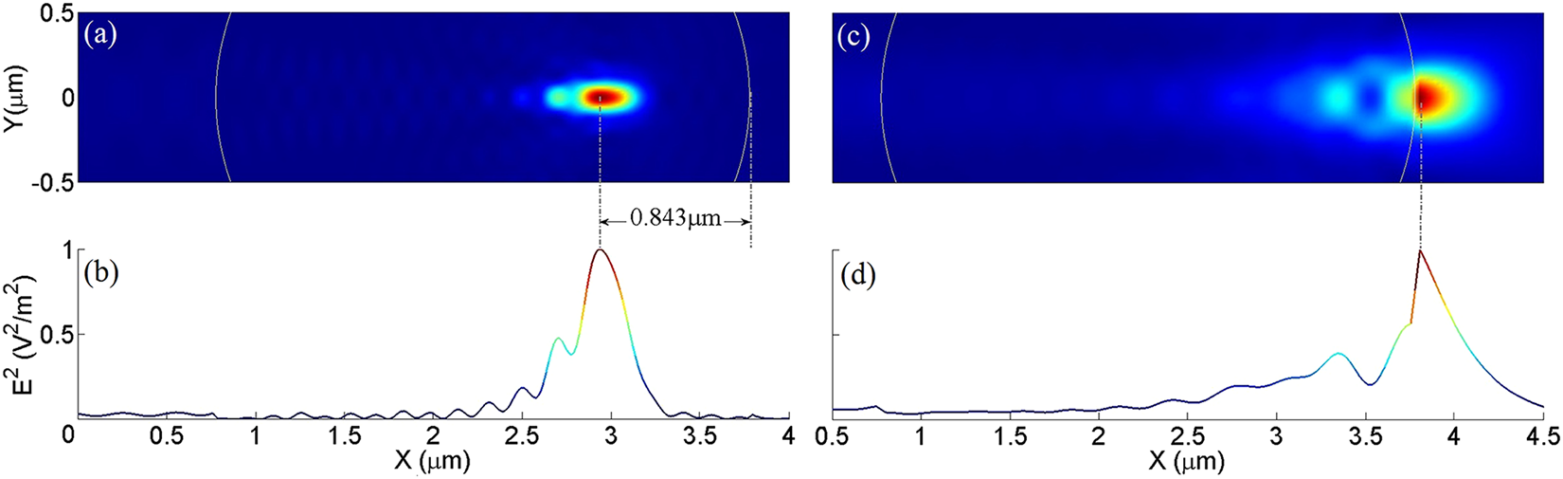
The electric intensity distribution (**a**,**b**) before and (**c**,**d**) after compensation of SAs. (**b**,**d**) are the electric intensities along the center line (i.e., *y* = 0) in (**a**,**c**), respectively. The parameters for calculation are: *n*_1_ = 1.34, *n*_2_ = 2.67 (**a**,**b**), *n*_2_ = 1.57 (**c**,**d**), *r* = 1.5 μm and λ_0_ = 0.69 μm. White curves indicate the front and rear edge of microsphere. Note that the electric intensity is normalized to unity.

In vector diffraction theories, the focal shift occurs in the optical system with small Fresnel number (FN) and the shift cannot be predicted by the classical vector diffraction theory of Richards and Wolf^23^ because of the invalidation of Debye approximation for small-Fresnel-number optical system^24^, and so does the optical system in the present article. Here, we defined the numerical aperture of microsphere as NA = *n*_1_ * sinα (see right panel of Fig. 1) just like ref. 12. Taking advantage of simple geometrical relationship presented in right panel of Fig. 1, we had α = 2 (*θ*_1_ − *θ*_2_), and NA_max_ = *n*_1_ * sinα_max_ for focusing at shadow side of microsphere. For legible illustration, we took a microsphere with radius *r* = 1.5 μm immerged in water (i.e., *n*_1_ = 1.34) as an example. Substituting *r*, *n*_1_ and λ_0_ ( =0.69 μm) into eq. (1), the refractive index of microsphere can be figured out as *n*_2_ = 2.67. Then, we had sin*θ*_1_ = 0.215λ_0_/*r* = 0.0989, sin*θ*_2_ = *n*_1_ * sin*θ*_1_/*n*_2_ = 0.0496, NA_max_ = 0.133. The electric intensity distribution in *xy* plane is plot in Fig. 2(a), and for better visualization we also plot the electric intensity along the center line, i.e., *y* = 0, in Fig. 2(b), from which it can be seen that the focus of incident needle is clearly shifted into the microsphere, about 0.843 μm away from the shadow side of microsphere, i.e., geometric focus.

To compensate the negative SAs, positive SAs should be enhanced. According to ref. 12, microsphere with smaller RIR has larger positive SAs (see Fig. 2 in ref. 12); we expect that properly small RIR will compensate the negative SAs. Therefore, the refractive index of microsphere should be reduced; eq. (1) can be modified as follow:

2 $${n}_{2}=2{n}_{1}\,\cos (0.215{\lambda }_{0}/2r)-{\rm{\Delta }}$$

where ∆ is the corrected value for enhancing positive SAs. Here, we determine the corrected value Δ by using FDTD method, which can represent the real physical scene as long as the space and time steps are fine enough and the simulation area is not too large. In FDTD Solutions (a commercial software), we determined the relationship between focal shift S and *n*_2_ by setting zero spherical aberration as target value and the refractive index of microsphere (i.e., *n*_2_) as variable, finding that when the refractive index of microsphere *n*_2_ equals to 1.57 the system possesses the smallest focal shift, near zero. That is, it is 1.57 the refractive index of microsphere *n*_2_ gets that the incident optical needle is actually focused at the shadow-side point B. Thus, the corrected value is found to be 1.1 for above example (i.e., microsphere with radius *r* = 1.5 μm immerged in water) when SAs are well eliminated. The electric intensity distribution in *xy* plane after SAs compensation is shown in Fig. 2(c), and the electric intensity along the center line in Fig. 2(d). In fact, the underlying physical mechanism for compensating SAs by reducing RIR is understandable from Snell’s law: for microsphere with fixed radius and surrounding medium (i.e., *θ*_1_ and *n*_1_ are unchangeable), the smaller *n*_2_ is, the larger *θ*_2_ is, i.e., the nearer the focus to the shadow side of microsphere is. Note we use the same definition of spherical aberration for microsphere as did in ref. 12.

By comprehensive analysis we found that if we define *h* = λ_0_/*r* as the relative aperture of microsphere eq. (2) can be written as follow:

3 $${n}_{2}=1.34(2-{(0.215h/2)}^{2}-0.825)$$

where $\cos \,x\approx 1-{x}^{2}/2$ was used for small value *x*. In eq. (3) the first two terms are the contribution of negative SAs that is an even function of relative aperture *h* and the last term is the contribution of positive SAs. Note that only primary SAs is taken into account for negative SAs. Another important fact is that the corrected value ∆ is proportional to the refractive index of surrounding medium (e.g., 1.34 in above example), and the scaling factor is 0.825. That is, eq. (3) could be generalized to any surrounding medium by replacing 1.34 with the refractive index of surrounding medium *n*_1_, such as free space, cedar wood oil, and deionized water and so on, i.e., the generalized formula can be expressed as:

4 $${n}_{2}={n}_{1}(2-{(0.215h/2)}^{2}-0.825)$$

The physical mechanism behind the generality is that if the refractive index of surrounding medium is scaled by η (i.e., *n*_1_ → η*n*_1_), the refractive index and radius of microsphere should be scaled by the same factor η (i.e., *n*_2_ → η*n*_2_, *r* → η*r*). Thus, the corrected value should be scaled by the same factor η to ensure that the relative aperture *h* and RIR remain unchanged, keeping the balance between the positive and the negative SAs. More detailed explanation will be given in parameter transformation of the Discussion section. we would like to point out that the eq. (4) is effective with the microsphere radius in the range from *n*_1_ * λ_0_ to 1.4 * *n*_1_ * λ_0_, larger radius will break the balance between the positive and negative SAs and the corrected value Δ is no longer constant but a function of radius.

## Discussion

In practical application, if one or more parameters are changed due to some restricted condition, how to determine the values of the remaining parameters without changing the system resolution. Here, eq. (4) will tell us the answer. To better illustrate the function of parameters transformation of eq. (4) Table 1 is given below. Normally, the issue of parameter transformation can be divided into three kinds case: I the illumination source and microsphere radius are altered while the surrounding medium remains unchanged; II the surrounding medium, the refractive index and radius of microsphere are changed while the illumination source remains unchanged; III all four parameters in eq. (4) are altered.

For case I, if the illumination wavelength is scaled by η (i.e., λ_0_ → ηλ_0_), the microsphere radius has to be scaled by the same factor η (i.e., *r* → η*r*) to ensure that the incident beam is still focused at the shadow side. For instance, the illumination wavelength in above example is not 0.690 μm but 0.405 μm (i.e., η = 0.587), and this is often the case for fluorescence microscope. Here, we call the illumination wavelength λ_0_ as active parameter, which has to be changed due to some restricted condition, and the microsphere radius *r* as passive parameter. The underlying physical mechanism is understandable according to eq. (4) that if the illumination wavelength λ_0_ and the microsphere radius *r* are scaled by the same factor η, then the relative aperture *h* in eq. (4) remains unchanged and so does RIR (i.e., *n*_2_/*n*_1_); the negative and positive SAs are not changed because they are related to relative aperture *h* and RIR, respectively; the SAs of microsphere after transformation is still well compensated and eq. (4) is satisfied. Thus, the incident beam will be focused at the shadow side.

Here, we explain case II in detail as it is a bit puzzling. In case II if the refractive index of surrounding medium is scaled by η (i.e., *n*_1_ → η*n*_1_), then the illumination wavelength in surrounding medium is scaled by 1/η (i.e., λ_0_ → λ_0_/η). We know that the width of incident beam is 0.43λ, λ is the wavelength in surrounding medium. In the case after transformation the width of incident beam should had be 0.43λ_0_/η. However, the width of incident needle is actual 0.43λ_0_ due to the normal incidence at the interface between air and the surrounding medium. In other words, the width of incident beam is expanded by η times. In order to keep the relative aperture *h* unchanged, the microsphere radius *r* should also be scaled by η times (i.e., *r* → η*r*). At the same time, if we scale the refractive index of microsphere by the same factor η (i.e., *n*_2_ → ηn_2_), then the relative index RIR and relative aperture *h* are both unchanged, the negative SAs and positive SAs remain well compensated. Thus, the incident beam will be focused at the shadow side of microsphere.

As for case III, it is just the combination of cases I and II. In this case there are two active parameters, the process of parameter transformation can be divided into two steps: First, the illumination wavelength is scaled by σ (i.e., λ_0_ → σλ_0_) the microsphere radius should be scaled by the same factor σ (i.e., *r* → σ*r*). Second, the refractive index of surrounding medium is scaled by η (i.e., *n*_1_ → η*n*_1_) the refractive index and radius of microsphere should also be scaled by the factor η (i.e., *n*_2_ → λ*n*_2_, *r* → ση*r*). Note that the order of these two steps has no effect on the performance of parameter transformation.

On the basis of eq. (4), not only can researchers setup an optical system of microsphere to focus the incident needle at the shadow side of microsphere with super-resolution, but they can also perform parameter transformation. Now we combine some concrete examples to demonstrate the above theory and illustrate the parameter transformation by eq. (4). All simulations were based on the FDTD Solutions (a commercial software). The Auto-uniform meshing with mesh accuracy 8, minimum mesh step of 0.25 nm and perfectly matched layer were used. For simplicity but not lose generality, the electric intensity in each case was normalized to unity.

**Figure 3:**
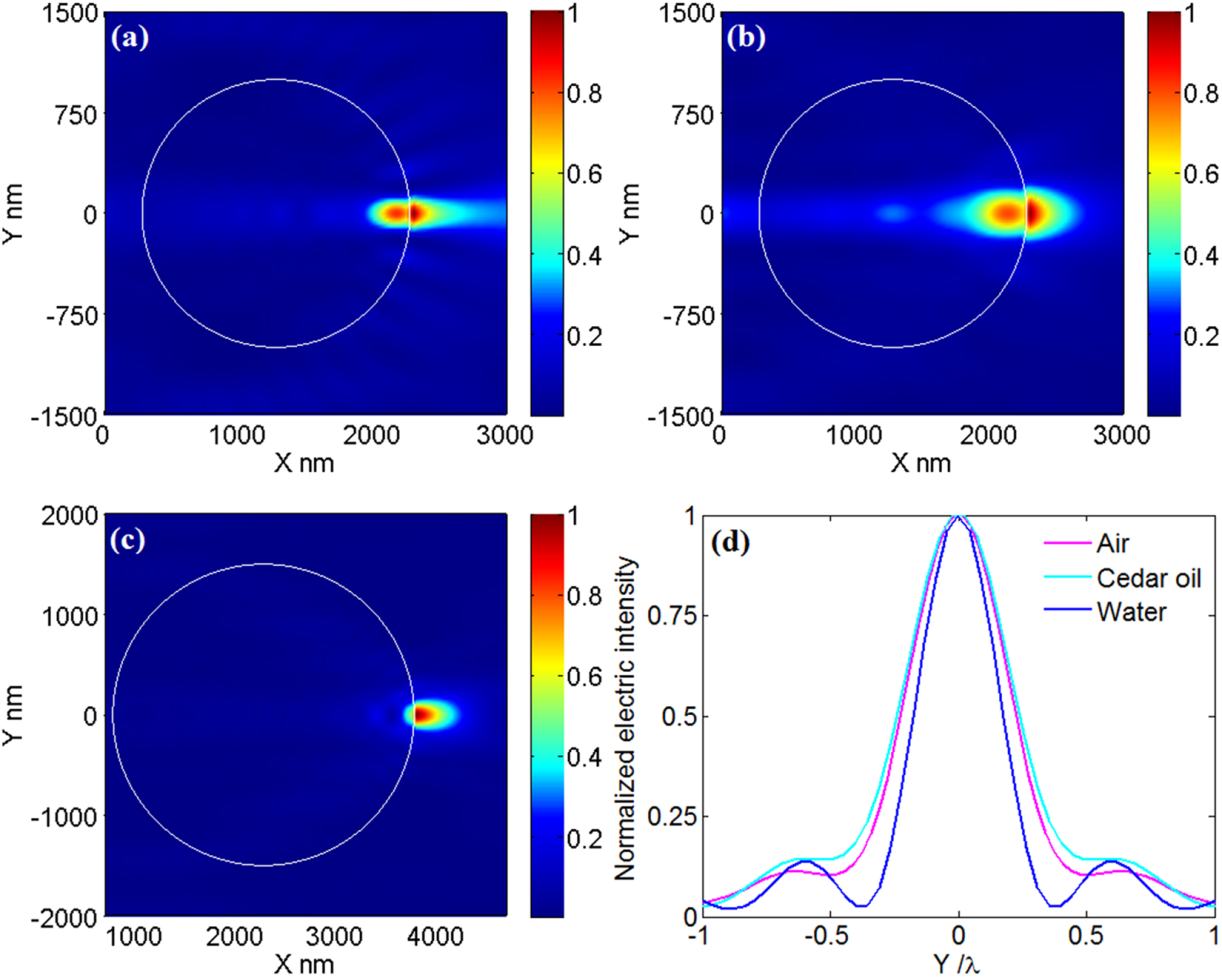
Electric intensity distribution around the microsphere immerged in (**a**) deionized water, (**b**) air, (**c**) cedar wood oil. The radii in (**a**–**c**) are 1 μm, 1 μm and 1.5 μm, respectively, and the electric intensity is normalized respectively to unity. (**d**) The transverse curves at the maximum value of electric intensity for (**a**–**c**). The horizontal axis is normalized respectively to the wavelength in surrounding medium, the same for the following.

First of all, we demonstrate the effectiveness of the theory compensating SAs, i.e., eq. (4). Here, we let the optical needle incident on microspheres with radii 1 μm, 1 μm and 1.5 μm, which were immerged in deionized water, air and cedar wood oil, respectively, as shown in Fig. 3(a–c), respectively. The illumination wavelength in free space is 0.69 μm. The refractive indices of microspheres were calculated by eq. (4). As expected the incident optical needles are all focused at the shadow-side surface in these three situations, where the full width at half-maximum (FWHM) of the focusing in these three situations are far below Abbe diffraction limit, and the FWHM in the case of deionized water is about 185.2 nm, even approximating 1/3 of the wavelength in water (i.e., λ_0_/3*n*_1_ = 171.6 nm). Above examples indicate that eq. (4) can be used to determine the parameters of microsphere system with high resolution.

**Figure 4:**
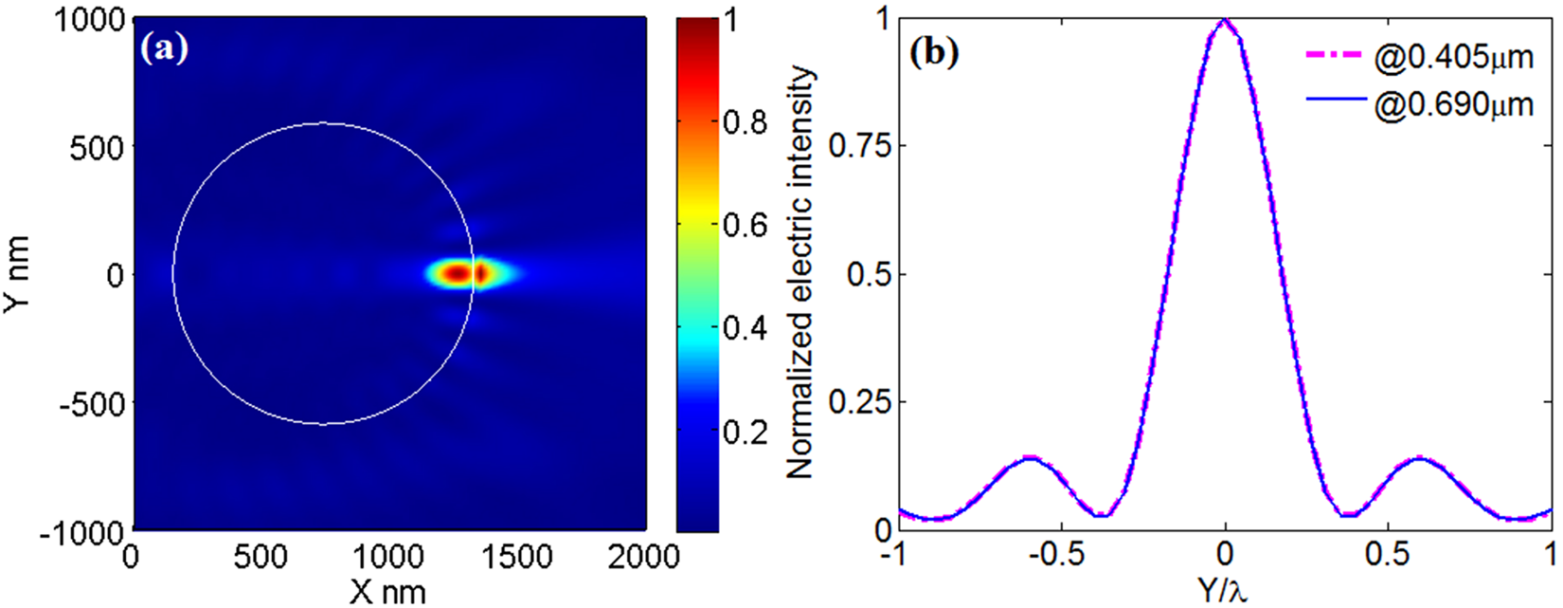
(**a**) Electric intensity distribution around the microsphere immerged in deionized water. The system parameters are: λ_0_ = 0.405 μm, *r* = 0.587 μm, *n*_1_ = 1.34, *n*_2_ = 1.56. (**b**) The transverse curves at the maximum value of electric intensity in Fig. 3(a) (blue) and in Fig. 4(a) (magenta).

**Figure 5:**
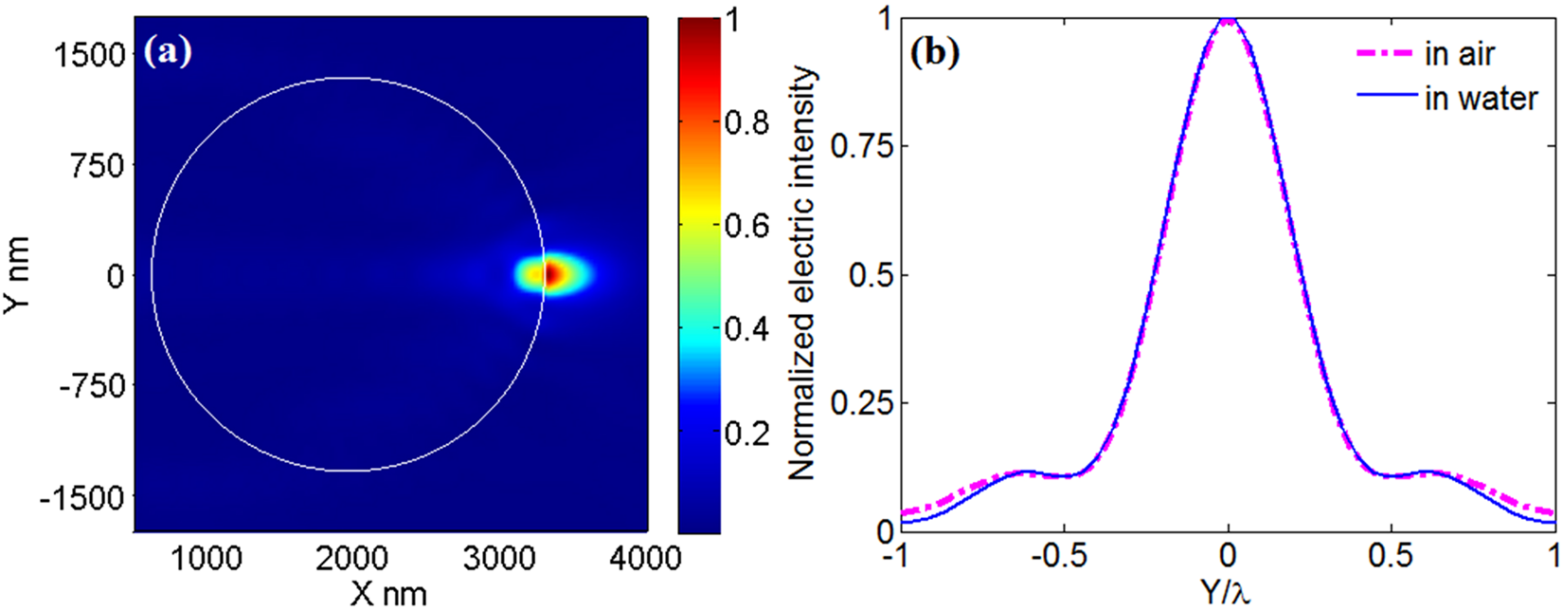
(**a**) Electric intensity distribution around the microsphere immerged in deionized water. The system parameters are: λ_0_ = 0.690 μm, *r* = 1.34 μm, *n*_1_ = 1.34, *n*_2_ = 1.57. (**b**) The transverse curves at the maximum value of electric intensity in Fig. 3(b) (magenta) and in Fig. 5(a) (blue).

**Figure 6:**
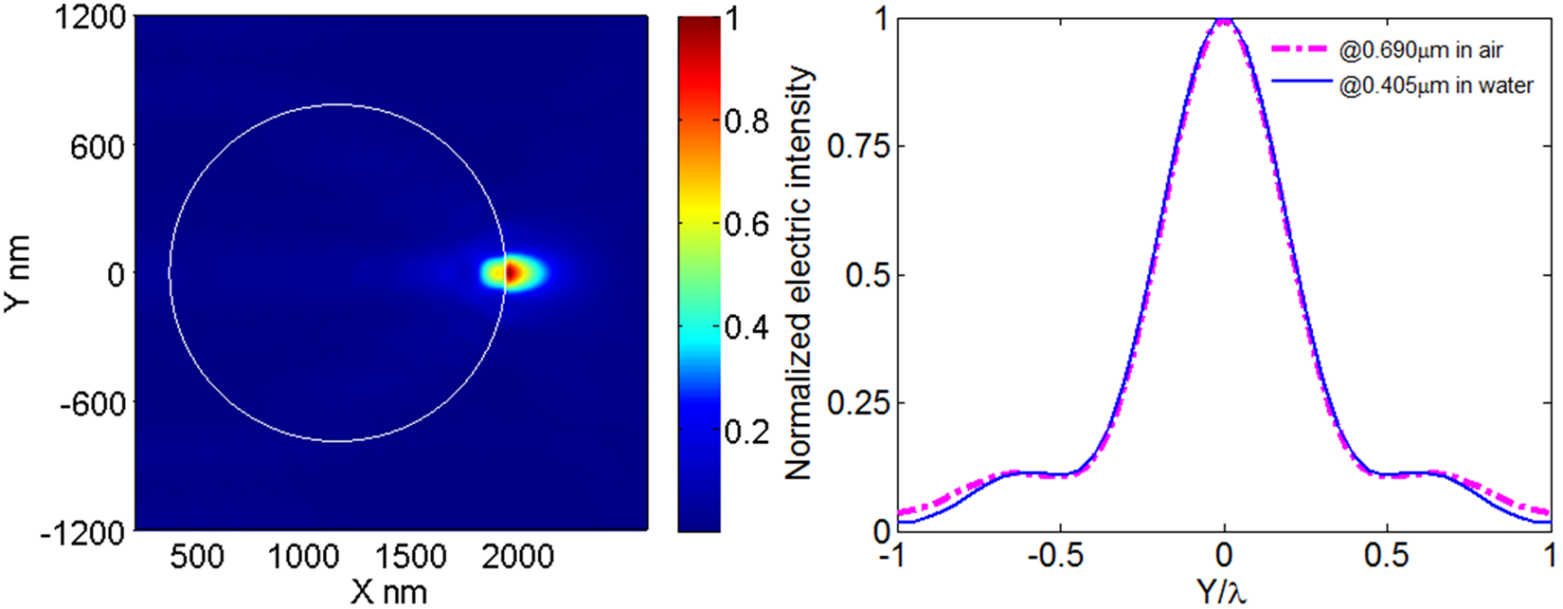
(**a**) Electric intensity distribution around the microsphere immerged in water. The system parameters are: λ_0_ = 0.405 μm, *r* = 0.786 μm, *n*_1_ = 1.34, *n*_2_ = 1.57. (**b**) The transverse curves at the maximum value of electric intensity in Fig. 3(b) (magenta) and in Fig. 6(a) (blue).

**Figure 7:**
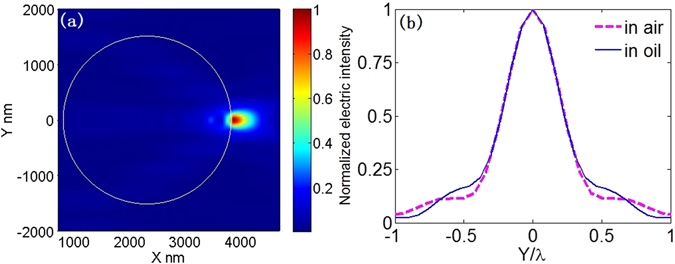
(**a**) Electric intensity distribution around the microsphere immerged in water. The system parameters are: λ_0_ = 0.69 μm, *r* = 1.515 μm, *n*_1_ = 1.515, *n*_2_ = 1.77. (**b**) The transverse curves at the maximum value of electric intensity in Fig. 3(b) (magenta) and in Fig. 7(a) (blue).

**Figure 8:**
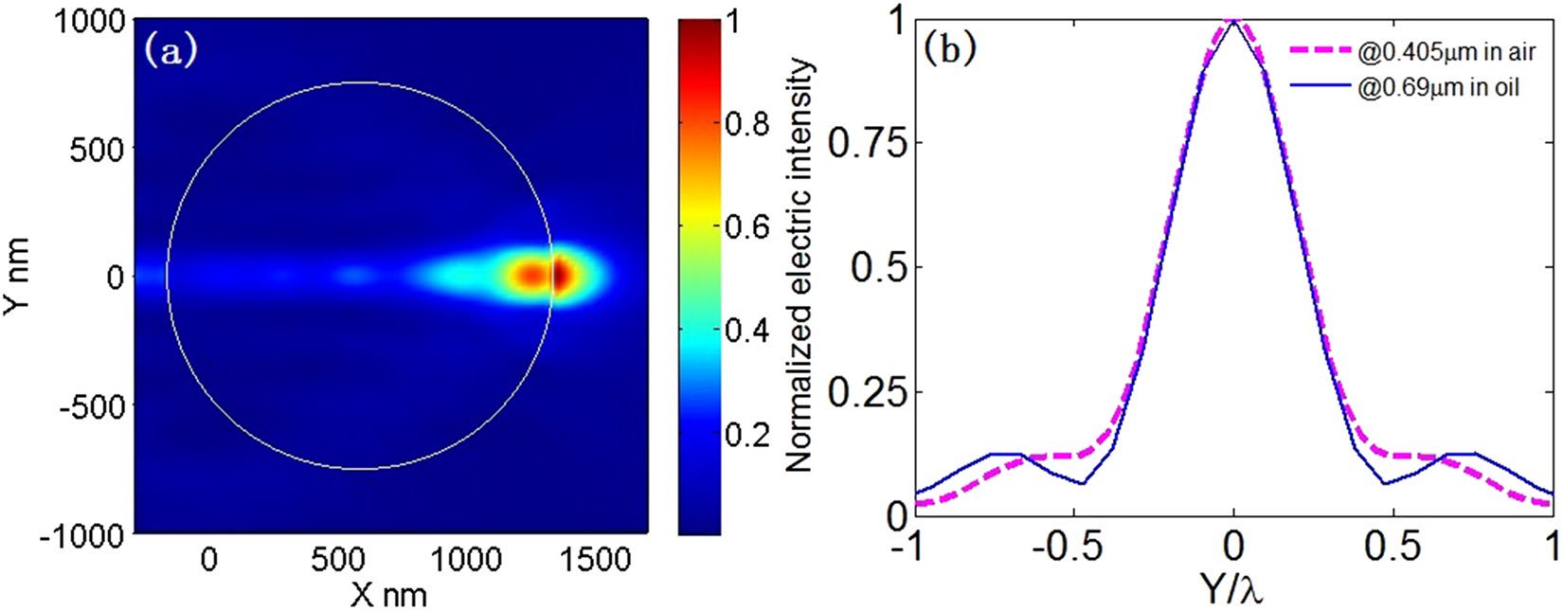
(**a**) Electric intensity distribution around the microsphere immerged in water. The system parameters are: λ_0_ = 0.69 μm, *r* = 1.515 μm, *n*_1_ = 1.515, *n*_2_ = 1.77. (**b**) The transverse curves at the maximum value of electric intensity in Fig. 3(b) (magenta) and in Fig. 7(a) (blue).

Now, to demonstrate that eq. (4) can be used to perform parameter transformation, we gave five examples for cases A–E, and the results are presented in Figs 4–8, respectively. For better understanding, the parameters before and after transformation for cases A–E are listed in Table 2. From Figs 4–8, it can be seen that according to the principles in Table 1 parameter transformation can be performed without changing the system resolution. Note the resolution in this paper is defined as to wavelength in surrounding medium.

**Table 2:** Parameters before and after transformation for Figs 4–8.

Notes		λ_0_ ( μm)	*n*_1_	*n*_2_	*r* ( μm)
A	before	0.69	1.34	1.56	1
	after	0.405^a^	1.34	1.56	0.587
B	before	0.69	1	1.17	1
	after	0.69	1.34^a^	1.57	1.34
C	before	0.69	1	1.17	1
	after	0.405^a^	1.34^a^	1.57	0.786
D	before	0.69	1	1.17	1
	after	0.69	1.515^a^	1.77	1.515
E	before	0.405	1	1.17	0.752
	after	0.69^a^	1.515^a^	1.77	1.5

^a^Denotes active parameter, others are passive parameters.

In conclusion we derived a simple but useful formula on the focusing of dielectric microsphere by eliminating the spherical aberrations of microsphere. On the basis of this formula, not only can researchers setup an optical system of microsphere with super-resolution to focus the incident needle at the shadow side of microsphere, but they can also perform parameter transformation. In order to facilitate the application, the principle of parameter transformation was summarized in Table 1. We would also like to point out that the formula in the present article is suitable for narrow incident beam, e.g., optical needle^19, 20^. For narrow incident beam, the performance of focusing of microsphere depends mainly on spherical aberration and rarely on numerical aperture of microsphere. While for wide incident beam, for instance plane wave, the effect of numerical aperture on the performance of focusing of microsphere should be taken into account. Our next step is trying to determine the similar formula for the illumination of plane wave or wide Gaussian beam and investigate the parameter transformation on the basis of that formula.

## Methods

All simulations were based on commercial software FDTD Solutions of Lumerical Solutions, Inc. In our model, the illumination source propagated along +*x* axis, and the source plane was located *x* = −2λ_0_ (λ_0_ is the illumination wavelength) and the focal plane was at *x* = 0. The simulation region was 8λ_0_ * 16λ_0_ * 16λ_0_, and the auto-uniform meshing with mesh accuracy 8 and minimum mesh step of 0.25 nm were used. The distance between source plane and the leftmost point of microsphere is the sum of illumination wavelength and microsphere radius. The whole simulation region was surrounded by a perfectly matched layer. Due to the absence of the optical needle in FDTD Solutions, we first calculated the six components of electric field (Ex, Ey, Ez) and magnetic field (Hx, Hy, Hz) at the transverse plane 2λ_0_ (i.e., *x* = −2λ_0_ in the formulas) ahead of the focal plane (i.e., *x* = 0 in the formulas) of 0.95-NA lens according to the formulas in ref. 20. To ensure the accuracy the transverse area for calculation was 16λ_0_ * 16λ_0_ that was divided into 800 * 800 meshes. Then, we imported the six components (Ex, Ey, Ez, Hx, Hy, Hz) into FDTD Solutions as illumination source.
